# Operation for Undescended Testicle and Hernia

**Published:** 1890-04

**Authors:** 


### OPERATION FOR UNDESCENDED TESTICLE AND HERNIA.

The patient was a baby ten months old, with good health, and suffering from no difficulty that the parents were aware of since birth. Within the past month the mother, while washing the child one morning, discovered an entire absence of the testis on the left side, and on the next morning that a very considerable enlargement was present. This condition of affairs lasted for a considerable length of time, and, finally, the child was brought here for diagnosis.

On examination it was found that the left testis was absent but there was some protrusion on the left side, which disappeared on raising the hips of the patient. The testis, it was believed, could be felt up within the inguinal canal.

The following operation was done in this case. An incision was made over the course of the cord, from the external ring well down along the antero-lateral surface of the scrotum, carrying it through the cellular tissue, cremaster muscle and intercolumnar facia, until the fibrous sheath of the cord was reached. The tesicle was then drawn down into the scrotum, and the canal obliterated by a double row of quilted sutures with the view of producing adhesive inflammation. In this way we prevent the reascent of the testis, and the descent of the intestines or omentum.

The wound was then closed by sutures, and dressed under the usual antiseptic precautions, but without drainage.

The operator stated that this was the second case of operation for hernia in a child of that age that had been done in this city, so far as he knew. The other case was operated upon at the Harlem Hospital two months previously, and the same steps of the operation were carried out as in this patient. The child made a good recovery, and the wound closed completely in ten days without any rise of temperature and with no constitutional disturbance whatever. This patient was also shown at the clinic.

